# Ectomycorrhizal fungi and past high CO_2_ atmospheres enhance mineral weathering through increased below-ground carbon-energy fluxes

**DOI:** 10.1098/rsbl.2014.0375

**Published:** 2014-07

**Authors:** Joe Quirk, Megan Y. Andrews, Jonathan R. Leake, Steve A. Banwart, David J. Beerling

**Affiliations:** 1 Department of Animal and Plant Sciences, University of Sheffield, Sheffield S10 2TN, UK; 2 Department of Soil Science, North Carolina State University, Raleigh, NC 27695, USA; 3 Kroto Research Institute, University of Sheffield, North Campus, Sheffield S3 7HQ, UK

**Keywords:** silicate weathering, mycorrhizal fungi, elevated carbon dioxide, photosynthate flux, ^14^CO_2_ tracer

## Abstract

Field studies indicate an intensification of mineral weathering with advancement from arbuscular mycorrhizal (AM) to later-evolving ectomycorrhizal (EM) fungal partners of gymnosperm and angiosperm trees. We test the hypothesis that this intensification is driven by increasing photosynthate carbon allocation to mycorrhizal mycelial networks using ^14^CO_2_-tracer experiments with representative tree–fungus mycorrhizal partnerships. Trees were grown in either a simulated past CO_2_ atmosphere (1500 ppm)—under which EM fungi evolved—or near-current CO_2_ (450 ppm). We report a direct linkage between photosynthate-energy fluxes from trees to EM and AM mycorrhizal mycelium and rates of calcium silicate weathering. Calcium dissolution rates halved for both AM and EM trees as CO_2_ fell from 1500 to 450 ppm, but silicate weathering by AM trees at high CO_2_ approached rates for EM trees at near-current CO_2_. Our findings provide mechanistic insights into the involvement of EM-associating forest trees in strengthening biological feedbacks on the geochemical carbon cycle that regulate atmospheric CO_2_ over millions of years.

## 1. Introduction

Approximately 20% of the contemporary terrestrial biosphere's annual primary production (*ca* 55−60 Gt C yr^−1^) [1] is allocated below-ground to support roots and associated symbiotic mycorrhizal fungi for nutrient and water acquisition [2]. In bioenergy terms, this flux equates to between 25 × 10^3^ and 83 × 10^3^ TW h of energy—up to six times annual electricity production from fossil fuels [3]—and plays an important role in driving global biogeochemical cycles. Conceptual advances and experimental evidence implicate increasing carbon-energy fluxes from trees to mycorrhizal fungi in accelerating inorganic nutrient cycling and land-to-ocean element transfers by enhancing mineral dissolution [4–8]. This biological feedback is important over multi-million-year timescales, because intensified weathering of continental calcium silicates strengthens the long-term sink for atmospheric CO_2_ ([CO_2_]_a_) by enhancing land-to-ocean calcium export and carbon sequestration into marine carbonates [5,9].

One mechanistic explanation for the proposed intensification of mineral weathering with the diversification of mycorrhizal fungi and their host trees is the ‘carbon-energy flux’ hypothesis. The hypothesis predicts that biotic weathering rates are driven by photosynthate-energy fluxes directed below-ground into mycorrhizal networks. These fluxes control the active surface area of mycorrhizal mycelium (the fungal network of hyphae) and its capacity for mineral weathering and inorganic nutrient acquisition for host trees [5], whose productivity is regulated by [CO_2_]_a_.

Here, we investigate the carbon-energy flux hypothesis in the context of the major evolutionary diversification of tree–fungus mycorrhizal partnerships, from ancestral arbuscular mycorrhizas (AM) to more recently evolved ectomycorrhiza (EM), and against the background of past high CO_2_ atmospheres. The origin and radiation of EM fungi began over 200 Myr after AM fungi in association with the evolutionary rise of angiosperm trees in the Cretaceous [10]. Studies of contemporary ecosystems indicate that EM fungal networks typically receive more (7–30%) net carbon fixed from their hosts than AM fungi (approx. 10%) [2], and this sustains larger EM mycelial networks [2,5–7]. Limited field evidence suggests that EM trees use this carbon flux to intensify weathering by a factor of 1.9–2.6 compared with AM trees, but this mechanistic linkage remains untested [7]. We quantify the effect of past high [CO_2_]_a_ because it regulates host tree productivity and likely ranged between 1100 and 1700 ppm during the Cretaceous when EM fungi first appeared [11,12]. Experiments were therefore undertaken at 1500 ppm and near-current 450 ppm [CO_2_]_a_ to capture the effect of past CO_2_-rich atmospheres compared with the modern situation.

**Figure 1.**
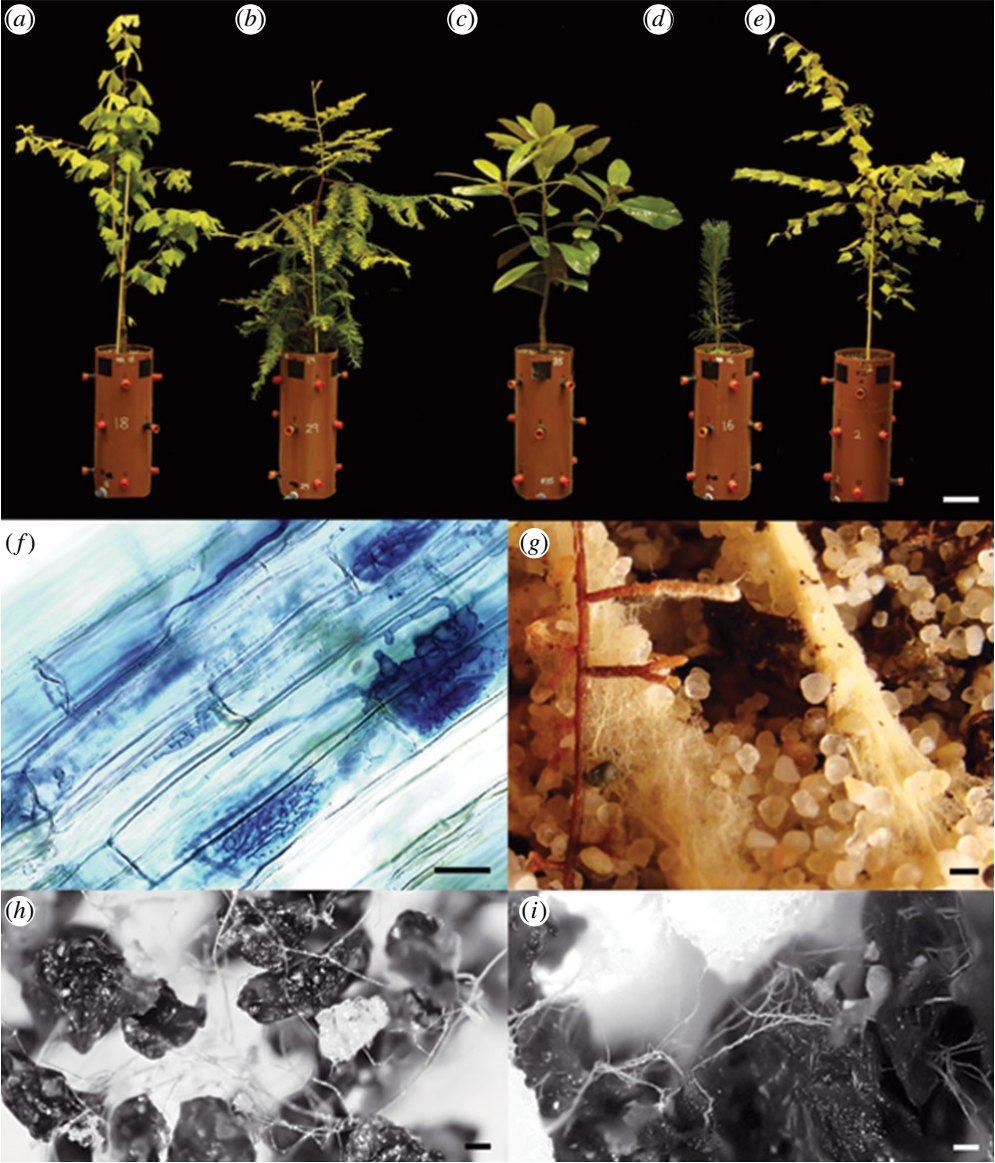
Weathering reactors with representative trees of (*a*) *G. biloba* (AM), (*b*) *S. sempervirens* (AM), (*c*) *M. grandiflora* (AM), (*d*) *P. sylvestris* (EM) and (*e*) *B. pendula* (EM) (scale bar, 100 mm). (*f*) Typical AM fungal colonization of *Ginkgo* roots and (*g*) EM hyphal tips and associated mycelium of *Pinus* roots from our experiments (scale bars, 1 mm). (*h*,*i*) Hyphal interactions with basalt grains in mesh cores (scale bars, 0.1 mm). (Online version in colour.)

Guided by time-constrained molecular phylogenies [13,14], we selected mycorrhizal host trees that represent exemplar taxa of past forests. These included early AM gymnosperm hosts *Ginkgo biloba* and *Sequoia sempervirens* and the early AM angiosperm host *Magnolia grandiflora* (figure 1). The responses of these AM partnerships were compared with *Pinus sylvestris* (EM gymnosperm) and *Betula pendula* (EM angiosperm), which have stem-group ages dating to the Cretaceous. We quantified carbon flows into mycorrhizal networks using standardized methodology involving ^14^CO_2_ tracers, and measured corresponding rates of calcium dissolution from basalt colonized by mycorrhizal mycelium as the most important silicate rock for global geochemical carbon cycling.

## 2. Material and methods

Saplings were cultivated in free-draining weathering reactors (figure 1*a*–*e*) in a sand and compost substrate mixed with species-specific mycorrhizal inoculum sourced from Westonbirt Arboretum, UK (see the electronic supplementary material). Saplings (*n* = 4) were kept alongside plant-free control reactors in controlled environment growth rooms (two per [CO_2_]_a_) and maintained at 450 or 1500 ppm [CO_2_]_a_ and otherwise constant environmental conditions. All weathering reactors were rotated between growth rooms each month and watered to field capacity twice weekly. We verified mycorrhizal status by light microscopy to visualize EM root tips and by clearing and staining roots to observe AM colonization (figure 1*f*,*g*).

Hyphal in-growth cores covered in root-excluding mesh (35 µm pore-size) were inserted horizontally into the weathering reactors at 200 mm depth (figure 1*a*–*e*). Cores were filled with 5.0 g of well-characterized Tertiary basalt (0.3–2.0 mm grain size, with a specific surface area of 68 cm^2^ g^−1^ [7]), along with 4.0 g of 0.05–0.10 mm pure quartz sand and sealed with gas-tight septa.

After five months, trees were pulse-labelled with 5 MBq ^14^CO_2_ liberated from NaH^14^CO_3_ into transparent polythene bags enclosing the canopy and sealed to the stem. Gas samples were taken at 2–5 h intervals from hyphal in-growth cores, post-labelling to monitor root and mycorrhizal fungal respiration of ^14^C. Cores were left *in situ* until the peak respired-^14^CO_2_ flux was detected, then removed to quantify non-respired-^14^C allocation to mycorrhizal hyphae colonizing basalt. Carbon allocation was calculated, accounting for the ^12^C : ^14^C ratio of CO_2_ inside the labelling bags in each treatment. We visually confirmed mycorrhizal hyphal colonization of basalt grains across all treatments (figure 1*h*,*i*). Core pore-water solution pH was measured and calcium silicate dissolution rates from basalt were determined relative to basalt samples from plant-free control reactors using sequential extractions following Quirk *et al*. [7] (see the electronic supplementary material).

Carbon fluxes to hyphal in-growth cores (following natural log normalization) and calcium dissolution rates were analysed using two-way ANOVA (mycorrhiza and [CO_2_]_a_ effects), both between and within mycorrhizal groupings using Minitab v. 12.21. We re-ran the two-way ANOVAs as ANCOVAs using natural log of calcium dissolution, with pH of core pore-water as a covariate to verify that calcium dissolution was not primarily driven by bulk pH.

## 3. Results and discussion

**Figure 2.**
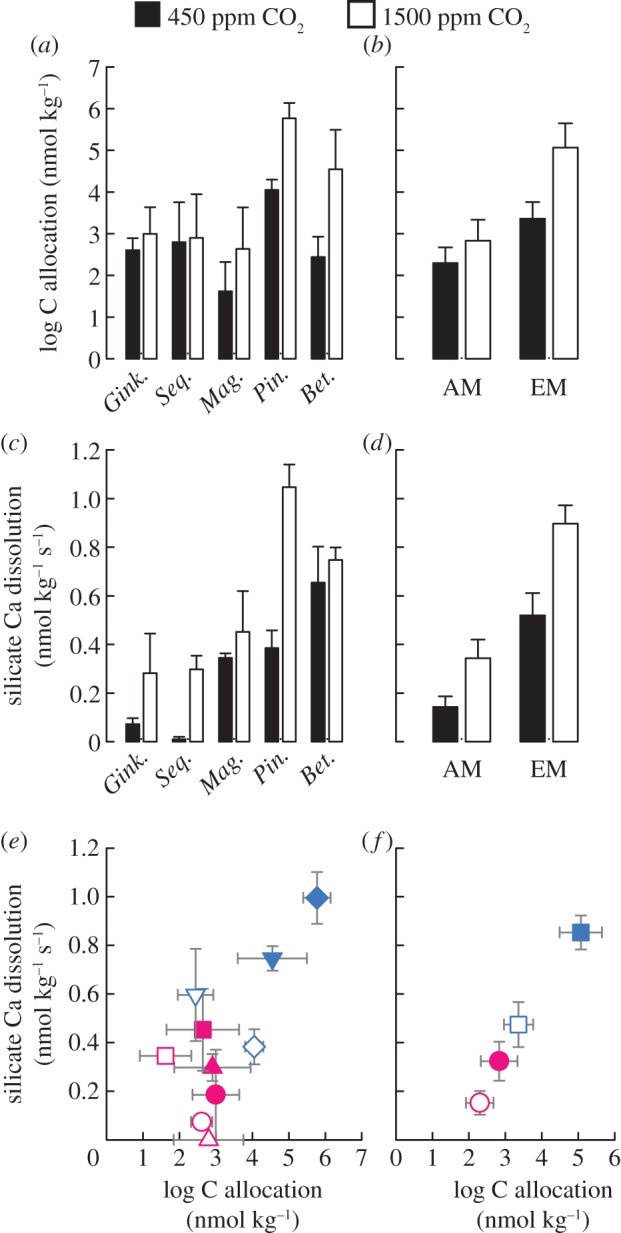
Photosynthate allocation through mycorrhizal mycelium to basalt and rates of silicate-bound calcium dissolution over the duration of the study for each tree species (*a*,*c*), and each mycorrhizal type (*b*,*d*) at each [CO_2_]_a_. Cross-plots of carbon allocation and silicate-bound calcium dissolution for (*e*) each species: AM *Ginkgo* (circles), AM *Sequoia* (triangles), AM *Magnolia* (squares), EM *Pinus* (inverted triangles) and EM *Betula* (diamond); and (*f*) mycorrhizal type (circles are AM and squares are EM). Open symbols represent 450 ppm, filled symbols represent 1500 ppm [CO_2_]_a_. All values show mean ± s.e.m. (Online version in colour.)

Our experiments show that both the advance from AM to EM mycorrhizal functional types and high atmospheric [CO_2_]_a_ increase carbon fluxes into mycorrhizal mycelium and drive enhanced silicate weathering (figure 2). At high [CO_2_]_a_, photosynthate allocation to mycorrhizal mycelium colonizing basalt was 2–7 times greater for EM *Pinus* and *Betula* than for the AM trees (figure 2*a*,*b*) (*F*_4,27_ = 3.84; *p* = 0.014). Moreover, this photosynthate allocation via EM mycelium doubled at 1500 ppm compared with that at 450 ppm [CO_2_]_a_ (figure 2*b*) (*F*_1,10_ = 9.07; *p* = 0.013). For AM trees, carbon allocation to mycorrhizal fungi varied greatly between species and generally doubled at 1500 ppm [CO_2_]_a_, but this was not significant (figure 2*a*,*b*) (*F*_1,18_ = 0.01; *p* = 0.920). Stimulation of carbon-energy flows into EM mycelium by high [CO_2_]_a_ is independently supported by previous studies of mycorrhizal *P. sylvestris* seedlings grown at 700 ppm versus 350 ppm [CO_2_]_a_ in which exudation of low molecular weight organic compounds, implicated in mineral weathering, increased by up to 270% [15].

In parallel with increased carbon allocation with the advance from AM to EM, and its response to high [CO_2_]_a_, there was a corresponding rise in rates of calcium silicate dissolution from basalt (figure 2*c*–*f*; electronic supplementary material, table S1) (two-way interaction: *F*_4,30_ = 3.18; *p* = 0.027). Calcium dissolution was not explained by the pH of solutions surrounding the basalt grains (*F*_1,29_ = 0.05; *p* = 0.825; log Ca-dissolution: *F*_1,29_ = 0.38; *p* = 0.543); and bulk soil solution pH associated with the basalt was close to neutral across all treatments (electronic supplementary material, table S2). Fungal-driven mineral dissolution is likely due to microscale acidification processes at the interface between fungal hyphal tips and the mineral surface, and such effects are not detected by bulk soil solution chemical analyses [5,16]. AM trees at high [CO_2_]_a_ showed a 2.4-fold increase in weathering compared with 450 ppm [CO_2_]_a_, and EM trees a 1.7-fold increase (figure 2*d*; *F*_1,36_ = 19.55; *p* < 0.0001), with both groups showing large variations between species (figure 2*c*).

Averaged across our tree–mycorrhiza partnerships, EM trees were associated with significantly higher calcium dissolution rates than AM species at both ambient and elevated [CO_2_]_a_ (*F*_1,36_ = 38.38; *p* < 0.0001) (figure 2*d*). EM-driven amplification of weathering at ambient [CO_2_]_a_ was 3.6 times that of AM, comparable with that seen for mature trees under field conditions [7]. Overall, silicate weathering rates by AM trees at high [CO_2_]_a_ approached those of EM trees at near-current [CO_2_]_a_ (figure 2*c*,*d*). This suggests that AM fungi, particularly under the high [CO_2_]_a_ characterizing much of the Phanerozoic, likely contributed more to terrestrial weathering processes, pedogenesis and biogeochemical element cycling than previously realized [17]. Greater investment of carbon in EM compared with AM mycelium is implicated in fuelling more carbon-intensive weathering processes. These include active uptake of weathered ions and acidification of the localized weathering environment by extrusion of organic acids and chelating compounds such as those complexing with calcium and aluminium [5,16].

Our results provide direct experimental support for the carbon-energy flux hypothesis and a unifying explanation for field observations of enhanced weathering by EM versus AM trees under common climates [6,7]. Although our experiments necessarily employed contemporary fungal strains, AM fungal genes controlling functions of the symbiosis are ancient and highly conserved, retaining similarities to fungi in the Mucoromycotina and Chytridomycota from which they diverged hundreds of millions of years ago [18]. Similarly, the ‘symbiotic toolbox’ genes of EM fungi—required for establishment and functioning of the symbiosis—probably date back tens of millions of years [19]. This indicates that our findings offer insights into the mechanisms behind strengthening terrestrial biotic feedbacks on the geochemical carbon cycle associated with evolutionary advancement of trees and mycorrhizal fungi. We propose that, in line with an earlier theoretical analysis [11], the spread of host trees partnering EM fungi increased below-ground carbon-energy fluxes, which accelerated silicate dissolution to play a role in driving the Earth's long-term [CO_2_]_a_ drawdown since the Cretaceous [11].

## Supplementary Material

JQuirk_C flux & silicate weathering_SuppInfo

## Data accessibility

Data used in this paper are presented in the electronic supplementary material, tables S1–S3.

## References

[1] Potter C Klooster S Genovese V 2012 Net primary production of terrestrial ecosystems from 2000 to 2009. Clim. Change 115, 365–378. (10.1007/s10584-012-0460-2)

[2] Leake J Johnson D Donnelly D Muckle G Boddy L Read D 2004 Networks of power and influence: the role of mycorrhizal mycelium in controlling plant communities and agroecosystem functioning. Can. J. Bot. 82, 1016–1045. (10.1139/b04-060)

[3] Key World Energy Statistics. 2011 International Energy Agency See http://www.iea.org/stats/index.asp.

[4] Brantley SL 2011 Twelve testable hypotheses on the geobiology of weathering. Geobiology 9, 140–165. 21231992 10.1111/j.1472-4669.2010.00264.x

[5] Taylor LL Leake JR Quirk J Hardy K Banwart SA Beerling DJ 2009 Biological weathering and the long-term carbon cycle: integrating mycorrhizal evolution and function into the current paradigm. Geobiology 7, 171–191. (10.1111/j.1472-4669.2009.00194.x) 19323695

[6] Koele N Dickie IA Blum JD Gleason JD de Graaf L 2014 Ecological significance of mineral weathering in ectomycorrhizal and arbuscular mycorrhizal ecosystems from a field-based comparison. Soil Biol. Biochem. 69, 63–70. (10.1016/j.soilbio.2013.10.041)

[7] Quirk J Beerling DJ Banwart SA Kakonyi G Romero-Gonzalez ME Leake JR 2012 Evolution of trees and mycorrhizal fungi intensifies silicate mineral weathering. Biol. Lett. 8, 1006–1011. (10.1098/rsbl.2012.0503) 22859556 PMC3497110

[8] Quirk J Leake JR Banwart SA Taylor LL Beerling DJ 2014 Weathering by tree-root-associating fungi diminishes under simulated Cenozoic atmospheric CO_2_ decline. Biogeosciences 11, 321–331. (10.5194/bg-11-321-2014)

[9] Berner RA 2004 The Phanerozoic carbon cycle: CO_2_ and O_2_. Oxford, UK: Oxford University Press.

[10] Brundrett MC 2002 Coevolution of roots and mycorrhizas of land plants. New Phytol. 154, 275–304. (10.1046/j.1469-8137.2002.00397.x) 33873429

[11] Taylor LL Banwart SA Leake JR Beerling DJ 2011 Modeling the evolutionary rise of ectomycorrhiza on sub-surface weathering environments and the geochemical carbon cycle. Am. J. Sci. 311, 369–403. (10.2475/05.2011.01)

[12] Fletcher BJ Brentnall SJ Anderson CW Berner RA Beerling DJ 2008 Atmospheric carbon dioxide linked with Mesozoic and early Cenozoic climate change. Nat. Geosci. 1, 43–48. (10.1038/ngeo.2007.29)

[13] Crisp MD Cook LG 2011 Cenozoic extinctions account for the low diversity of extant gymnosperms compared with angiosperms. New Phytol. 192, 997–1009. (10.1111/j.1469-8137.2011.03862.x) 21895664

[14] Wikström N Savolainen V Chase MW 2001 Evolution of the angiosperms: calibrating the family tree. Proc. R. Soc. Lond. B 268, 2211–2220. (10.1098/rspb.2001.1782) PMC1088868 11674868

[15] Johansson EM Fransson PMA Finlay RD van Hees PAW 2009 Quantitative analysis of soluble exudates produced by ectomycorrhizal roots as a response to ambient and elevated CO_2_. Soil Biol. Biochem. 41, 1111–1116. (10.1016/j.soilbio.2009.02.016)

[16] Landeweert R Hoffland E Finlay RD Kuyper TW van Breemen N 2001 Linking plants to rocks: ectomycorrhizal fungi mobilize nutrients from minerals. Trends Ecol. Evol. 16, 248–254. (10.1016/S0169-5347(01)02122-X) 11301154

[17] Lambers H Mougel C Jaillard B Hinsinger P 2009 Plant–microbe–soil interactions in the rhizosphere: an evolutionary perspective. Plant Soil 321, 83–115. (10.1007/s11104-009-0042-x)

[18] Tisserant E 2013 Genome of an arbuscular mycorrhizal fungus provides insight into the oldest plant symbiosis. Proc. Natl Acad. Sci. USA 110, 20 117–20 122. (10.1073/pnas.1313452110) PMC3864322 24277808

[19] Hibbett DS Matheny BP 2009 The relative ages of ectomycorrhizal mushrooms and their plant hosts estimated using Bayesian relaxed molecular clock analyses. BMC Biol. 7, 1–13. (10.1186/1741-7007-7-13) 19284559 PMC2660285

