# Supplementary material for: Ectomycorrhizal fungi and past high CO2 atmospheres enhance mineral weathering through increased below-ground carbon-energy fluxes
Source: Biol Lett. 2014 Jul;10(7):20140375. doi: 10.1098/rsbl.2014.0375 (PMC4126629; doi:10.1098/rsbl.2014.0375)
Supplement: JQuirk_C flux & silicate weathering_SuppInfo [file rsbl20140375supp1.pdf]

**Electronic Supporting Materials (ESM)**

# Ectomycorrhizal fungi and past high $CO_2$ atmospheres enhance mineral weathering through increased belowground carbon-energy fluxes

Joe Quirk*, Megan Y. Andrews, Jonathan R. Leake, Steve A. Banwart and David J. Beerling

**Author for correspondence (j.quirk@sheffield.ac.uk)*

## Detailed Methods

One-to-two year-old saplings of *Ginkgo biloba* (L.), *Sequoia sempervirens* (D. Don. [Endl.]), *Magnolia grandiflora* (L.), *Pinus sylvestris* (L.) and *Betula pendula* (Roth) were sourced from UK nurseries. The saplings were cultivated in specially designed 160 mm-diameter polyvinylchloride (PVC) free-draining weathering reactors (figure 1). The substrate consisted of 1:1 by volume Chelmsford '16/30' sand (WBB Minerals, Cheshire, UK) and potting compost (Levington M3, Scotts, Ipswich, UK) mixed with ca.0.5% by volume species-specific mycorrhizal inoculum consisting of fresh root material from beneath established stands of our respective species at the UK National Arboretum, Westonbirt, UK. Trees ($n$ = 4) were kept alongside plant-free control reactors in replicated ($n$ = 2 per $[CO_2]_a$) controlled-environment growth rooms (Conviron, Controlled Environments Ltd. Manitoba, Canada) and maintained at 450 ppm or 1500 ppm $[CO_2]_a$ and otherwise constant growth conditions of 80% relative humidity, 18/20°C night/day cycle with a 14-h photoperiod and canopy light intensity of 500 µmol $m^{-2}$ $s^{-1}$ for the entirety of the photoperiod and duration of the experiment. Trees were rotated between growth rooms on a monthly basis to avoid potential block effects and were well-watered with reverse-osmosis water twice weekly. We verified the mycorrhizal status of our saplings by light microscopy to visualise ectomycorrhizal (EM) root tips and by clearing and staining roots to observe arbuscular mycorrhizal (AM) colonisation.

Hyphal in-growth cores constructed from 85 mm-length PVC pipe (13.4 mm internal diameter) with two windows cut into the sides (ca. 2500 $mm^2$ total window area) covered in root-excluding mesh (35 µm-pore-size woven nylon; Plastok Associates Ltd. Birkenhead,

Wirral, UK) were inserted horizontally into ports at 200 mm depth on the side of the weathering reactors and sealed in place with rubber O-rings. The outer ends of the hyphal in-growth cores were sealed with gas-tight rubber septa (Suba-Seal, Sigma-Aldrich, Dorset, UK) allowing gas sampling from within the cores. The cores were filled with 5.0 g of well-characterised Tertiary basalt grains from Northern Ireland (described in Quirk *et al.* 2012) (0.3–2.0 mm grain size) along with 4.0 g of 0.05–0.10 mm pure quartz sand (Multi-Lab QuartzTec Ltd., East Kilbride, UK). Brunauer-Emmett-Teller (BET) analysis using a Gemini V2365 system (Micromeritics Instrument Corp., Georgia, USA) was used to obtain the surface area of the basalt. Samples were degassed at 22°C for at least 16 hours. BET measurements were performed using $N_2$ as analysis adsorbate. The basalt in our grain size fraction had a surface area of 68 $cm^2$ $g^{-1}$.

After five months' growth, trees were pulse-labelled for 4–6 h with 5 MBq $^{14}C$-$CO_2$ liberated from 67.5 µl $NaH^{14}CO_3$ using 1 ml 25% *(v:v)* lactic acid into 58-L transparent polythene bags enclosing the entire canopy of each tree and sealed to the stem with insulation tape. Gas samples (5 ml) were taken from the airspace inside the hyphal in-growth cores at 2-5-h intervals over the following 160 h to monitor root and mycorrhizosphere respiration of $^{14}C$, an indicator of canopy-fixed C-allocation via mycorrhizal networks into the cores. Radioactivity of the gas samples was measured by injecting the $^{14}CO_2$-containing air samples through a gas-tight septum into Carbosorb (Perkin Elmer, Beaconsfield, UK) to trap $CO_2$ and then mixed with Permafluor scintillant (Perkin Elmer) (10mL:10mL ). Radioactivity was measured via liquid scintillation counting (Packard Tri-carb 3100TR, Isotech, Chesterfield, Derbyshire, UK) using quench calibrations and unlabelled controls to determine background counts, which were subtracted. Hyphal in-growth cores were left *in situ* until peak respired $^{14}C$ flux was detected, at which point cores were removed from the reactors, frozen and then lyophilised. The $^{14}C$ allocation (in the form of microbial biomass – including mycorrhizal hyphae – exudates and precipitates) to the rock grains was then quantified via sample oxidation (100 mg rock grains) (Packard Sample Oxidizer 307; Isotech) and liquid scintillation counting of released $^{14}CO_2$ trapped in Carbosorb (Perkin Elmer) mixed with Permafluor scintillant (Perkin Elmer). Total allocation of photosynthate carbon was calculated in each case adjusting for the $^{12}CO_2$:$^{14}CO_2$ ratio of the air inside the labelling bags at 450 ppm and 1500 ppm $[CO_2]_a$. For subsamples from all species, we visually confirmed, but did not formally quantify, mycorrhizal hyphal colonization of basalt grains in the cores for all treatments.

We assessed physico-chemical alteration of the basalt grains within the hyphal in-growth cores at the end of the experiment using a sequential chemical extraction (described

previously in Quirk *et al.* 2012 and references therein) allowing assessment of elemental dissolution from silicate minerals in the basalt over the duration of the experiment. The extractions involved removal of the water-extractable, exchangeable, (1M ammonium acetate), carbonate (1M sodium acetate and acetic acid, pH 5.0) and oxide fractions (0.5M hydroxylamine-hydrochloride in 25% acetic acid followed by 0.1M ammonium oxalate adjusted to pH 3.0 with 0.2M oxalic acid and 0.1M ascorbic acid) of the basalt grains. Previous analyses demonstrate that following the extraction of the exchangeable and carbonate phases, the remaining calcium extracted within the oxide fractions thereafter is derived from silicate minerals within the basalt (Quirk *et al.* 2012; *Biol. Letts.* **8**, 1006-1011). Extraction solutions were diluted, acidified with 1% nitric acid and calcium concentrations of the oxide fraction were determined using inductively coupled plasma mass spectrometry (PerkinElmer Elan DRC II, MA, USA). We used the water extractable fraction to assess pH of the pore-water solutions to verify that calcium dissolution from the basalt was not primarily driven by pH effects within the cores.

Calcium dissolution from basalt was estimated for each treatment relative to basalt samples taken from plant-free control reactors using: Ca dissolution (nmol $kg^{-1}$ $s^{-1}$) = $(Ca_{con} - Ca_{tree})/time$; where $Ca_{con}$ is the amount of Ca extracted from the silicates in the basalt from plant-free control reactors (nmol $kg^{-1}$), $Ca_{tree}$ is the amount of Ca extracted from basalt in each replicate treatment (nmol $kg^{-1}$) and *time* is the duration of the experiment (13,564,800 s). Comparison of the amount of calcium extractable from the silicate phase of freshly prepared, unreacted basalt grains with that from the plant-free, AM and EM tree treatment indicates a calcium weathering intensification of 6 – 22% from unreacted to plant-free controls, followed by a further 21 – 24% by AM trees and 39 – 43% by EM trees (Table S3).

Statistical testing was conducted using MINITAB v.12.21. The allocation of total organic carbon flux to hyphal in-growth cores, following natural log transformation to satisfy assumptions of homogeneity of variance, and calcium dissolution rates were analysed using two-way ANOVA (tree-mycorrhiza partnerships and $[CO_2]_a$ effects), both between and within mycorrhizal groupings. We also re-ran the two-way ANOVAs as ANCOVAs using logged calcium dissolution data with the pH of core pore-water samples as a covariate to verify that calcium dissolution was not primarily driven by bulk solution pH.

**Table S1.** $^{14}C$ allocation into hyphal in-growth cores (nmol $kg^{-1}$ ± s.e.m.)

| | 450 ppm $[CO_2]_a$ | 1500 ppm $[CO_2]_a$ |
|---|---|---|
| *Ginkgo* (AM) | 2.61 ± 0.29 | 3.00 ± 0.64 |
| *Sequoia* (AM) | 2.80 ± 0.96 | 2.90 ± 1.05 |
| *Magnolia* (AM) | 1.62 ± 0.70 | 2.64 ± 1.00 |
| *Pinus* (EM) | 4.05 ± 0.25 | 5.77 ± 0.37 |
| *Betula* (EM) | 2.44 ± 0.49 | 4.55 ± 0.95 |

**Table S2.** Basalt core pore-water pH (± s.e.m.)

| | 450 ppm $[CO_2]_a$ | 1500 ppm $[CO_2]_a$ |
|---|---|---|
| *Ginkgo* (AM) | 6.47 ± 0.09 | 6.53 ± 0.08 |
| *Sequoia* (AM) | 6.43 ± 0.16 | 6.59 ± 0.11 |
| *Magnolia* (AM) | 6.66 ± 0.08 | 6.73 ± 0.04 |
| *Pinus* (EM) | 6.71 ± 0.07 | 6.71 ± 0.11 |
| *Betula* (EM) | 6.61 ± 0.07 | 6.28 ± 0.26 |
| Plant-free | 6.69 ± 0.08 | 6.70 ± 0.09 |

**Table S3.** Silicate bound Ca chemically extracted from basalt grains at the end of the experiment (μmol $g^{-1}$ ± s.e.m.) ($n = 4$). Ca dissolution over the course of the experiment is calculated based on the difference between plant-free controls and tree treatments.

| | 450 ppm $[CO_2]_a$ | 1500 ppm $[CO_2]_a$ |
|---|---|---|
| *Ginkgo* (AM) | 25.08 ± 0.79 | 28.74 ± 3.09 |
| *Sequoia* (AM) | 29.12 ± 1.48 | 27.03 ± 0.75 |
| *Magnolia* (AM) | 20.95 ± 0.27 | 22.85 ± 1.01 |
| *Pinus* (EM) | 20.43 ± 0.98 | 16.83 ± 1.26 |
| *Betula* (EM) | 19.37 ± 2.44 | 20.94 ± 0.68 |
| Plant-free | 25.63 ± 2.42 | 31.06 ± 4.75 |
| Unreacted basalt* | 33.14 ± 1.28 | |

* Freshly prepared basalt grains that were not buried in weathering reactors
